# Comparative evaluation of intermediate solutions in prevention of brown precipitate formed from sodium hypochlorite and chlorhexidine gluconate

**DOI:** 10.1002/cre2.654

**Published:** 2022-09-14

**Authors:** Vashti Bueso, Neha Parikh, Tanguy Terlier, Julian N. Holland, Nima D. Sarmast, Ji Wook Jeong

**Affiliations:** ^1^ School of Dentistry The University of Texas Health Science Center at Houston Houston Texas USA; ^2^ Department of Diagnostic and Biomedical Sciences, The University of Texas Health Science Center at Houston School of Dentistry Houston Texas USA; ^3^ SIMS Laboratory, Shared Equipment Authority Rice University Houston Texas USA; ^4^ Office of Research, The University of Texas Health Science Center at Houston School of Dentistry Houston Texas USA; ^5^ Department of Endodontics, The University of Texas Health Science Center at Houston School of Dentistry Houston Texas USA

**Keywords:** brown precipitation, chlorohexidine gluconate, intermediate irrigation, sodium hypochlorite, sodium thiosulphate

## Abstract

**Objectives:**

To evaluate intermediate treatments between sodium hypochlorite and chlorhexidine gluconate irrigations for the prevention of a toxic brown precipitate in root canal therapy.

**Materials and Methods:**

Thirty‐nine premolars were irrigated with 6% sodium hypochlorite and divided into either: No intermediate treatment; Dry paper points; three different irrigations with 17% ethylenediaminetetraacetic acid, deionized water, or 5% sodium thiosulfate. 2% chlorhexidine gluconate was the final irrigant in all groups. Sectioned teeth were analyzed for brown precipitate intensity and area using stereomicroscopy and components related to para‐chloroaniline using Time‐of‐Flight Secondary Ion Mass Spectrometry (ToF‐SIMS).

**Results:**

Stereomicroscopy showed that 5% STS significantly reduced brown precipitate intensity and area as compared with no intermediate irrigation (*p* < .05, Chi‐square, generalized linear model, and Tukey's multiple comparison tests). Utilizing ToF‐SIMS, 5% sodium thiosulfate was most effective in reducing the components representing para‐chloroaniline and chlorhexidine gluconate.

**Conclusion:**

The 5% sodium thiosulfate was most effective among other intermediate treatments, assessed by stereomicroscopy and ToF‐SIMS.

## 1 INTRODUCTION

Sodium hypochlorite (NaOCl, 0.5%–6%) solution with significant antimicrobial activity is the most widely used irrigant that also dissolves organic debris in the canal system (Naenni et al., 2004). Chlorhexidine gluconate (CHX, 0.12%–2%) with antiseptic properties is viewed as a safer alternative to NaOCl (Yesilsoy et al., 1995) although it does not dissolve organic tissues (Naenni et al., 2004). Because of their distinct complementary properties, CHX and NaOCl are sometimes used for irrigation in the same tooth. However, consecutive use of CHX and NaOCl results in the formation of orangish brown precipitation that can interfere with the root canal obturation (Bui et al., 2008; Zehnder, 2006). Additionally, the chemical composition of the brown precipitate (BP) is reported to be similar or identical to that of para‐chloroaniline, which is a known carcinogen and genotoxic substance (Basrani et al., 2007, 2010; Chhabra et al., 1991). Recent studies also report BP‐induced cytotoxicity in eukaryotic model systems (Cintra et al., 2014; Jeong et al., 2021; Nocca et al., 2017; Patil et al., 2016). Although modified irrigation protocols have been suggested, including 70% isopropyl alcohol, 3.86% sodium thiosulfate, 6.25% sodium metabisulfite, 14% ethylenediaminetetraacetic acid (EDTA), 50% citric acid, and sterile saline (Chhabra et al., 2018; Krishnamurthy & Sudhakaran, 2010; Mortenson et al., 2012), there is no consensus on any specific intermediate irrigation protocol that would completely eliminate the BP formation (Chhabra et al., 2018; Krishnamurthy & Sudhakaran, 2010; Mortenson et al., 2012). The mechanism of eliminating BP by the majority of proposed intermediate irrigants between NaOCl and CHX solutions is also unclear. Further, there are limited studies assessing the comparative efficacy of these intermediate irrigants in preventing BP. One potential mechanism to prevent BP formation is by neutralizing NaOCl. EDTA neutralizes NaOCl by chelating free chlorine, lending to its interest as a potential agent to prevent BP (Grawehr et al., 2003). Sodium thiosulfate (STS; Na_2_S_2_O_3_) is another potential intermediate irrigant with antioxidative properties that neutralizes NaOCl (Chhabra et al., 2018; Hegde et al., 2012; Sariyilmaz et al., 2019). In endodontics, STS was first suggested as an irrigant solution in 1966 and is known to inactivate halogens such as chlorine (Möller, 1966). Because NaOCl irrigation lowers the bonding strength of dentine to composite resin, STS can be used to recover adhesive strength to dentine for composite resin (Pimentel Corrêa et al., 2016). Recently, 5% STS was effective in clearing BP from bovine teeth (Alberto et al., 2021). However, the study only used a visual examination of BP. Another study testing 3.86% STS did not significantly reduce BP in extracted human teeth (Chhabra et al., 2018). Simpler approaches such as intermediate irrigation with water or intermediate drying with paper points are relevant for evaluation in comparison to NaOCl neutralizing intermediate irrigants.

In this study, the comparative efficacy of intermediate irrigation with deionized water, 17% EDTA, 5% STS or drying with paper points was evaluated for preventing BP using extracted human premolars. Stereomicroscopy and Time‐of‐Flight Secondary Ion Mass Spectrometry (ToF‐SIMS) were utilized to assess the presence and chemical composition of BP. We hypothesized that an intermediate irrigation with 5% STS, 17% EDTA, deionized water, or drying with paper points will reduce the BP formation with descending efficacy respectively during root canal irrigation.

## 2 MATERIALS AND METHODS

### 2.1 Ethics

Experiments in this study were conducted in compliance with institutional guidelines. This study was exempted by the committee for the protection of human subjects (HSC‐DB‐19‐0032).

### 2.2 Ex vivo tooth assay

Ex vivo teeth stored in 10% formalin solution for 2 weeks followed by a 1:10 solution of diluted bleach for over 3 months, until the day of the experiment, were used for the study. Based on previously published articles with comparable study designs (Bui et al., 2008; Chhabra et al., 2018; Krishnamurthy & Sudhakaran, 2010; Mortenson et al., 2012), consultation with a statistician (NH), and the limited number of available human teeth, the final sample size was 39, fully developed extracted single‐rooted human premolars. Periapical radiographs were taken in buccolingual and mesiodistal planes of maxillary and mandibular premolar teeth, selecting for a single oval‐shaped canal morphology. All teeth were endodontically accessed and shaped to a final apical diameter of 35/0.04 using Vortex Blue rotary files (Dentsply, USA). A #10/0.02 K‐file (Dentsply Maillefer) was used to establish patency in each canal before irrigation. Working length was established by subtracting 0.5 mm from the length at which a #10 K‐file could be seen at the apical foramen. The 6% NaOCl (VISTA™, USA) was used for initial irrigation during the instrumentation. 3 ml of the 6% NaOCl was delivered at a steady rate of  approximately 4 ml per minute through a 27‐gauge side vented irrigation probe positioned at a depth within 3 mm of the apex. 3 ml NaOCl was used to irrigate after each rotary file for a total of 12 ml during instrumentation. All teeth were thereafter randomly distributed into five groups (Groups 1–5). Based on limited samples and clear preliminary results of the pilot study, the number of teeth in Groups 1 and 2 was limited to 5. Ten teeth each were randomly assigned to Groups 3–5. However, one tooth in Group 5 was damaged post intermediate irrigation and was eliminated from further analysis. The final numbers and treatment of teeth were as follows:

- Group 1: Control group with no intermediate irrigation or treatment (*N* = 5).
- Group 2: No intermediate irrigation but the canals were dried with paper points (Brasseler, GA, USA) after 6% NaOCl (*N* = 5).
- Group 3: Intermediate irrigation for 1 min by 3 ml of 17% EDTA (VISTA™, USA) (*N* = 10).
- Group 4: Intermediate irrigation for 1 min by 3 ml of deionized water (*N* = 10).
- Group 5: Intermediate irrigation for 1 min by 3 ml of 5% STS (Cesco, TX, USA) prepared w/v in deionized water (*N* = 9).

All groups were finally irrigated with 3 ml of 2% CHX (Brasseler, GA, USA) at a steady rate of approximately 4 ml per minute through a 27‐gauge side vented irrigation syringe positioned at a depth within 3 mm of the apex. Groups 2–5 were dried with paper points. After the final irrigation, the teeth were laid on their sides and decoronated using a double‐sided, medium‐sized hyperflex diamond disc (Brasseler, GA, USA) with a slow‐speed (25,000 rpm) handpiece at the cementoenamel junction. Each root was subsequently split longitudinally in the buccolingual direction. Sectioned teeth were stored with sectioned‐side face up on strips of rope wax in airtight boxes until further analysis. Each canal was investigated at 10–40X magnification using a stereomicroscope (Nikon SMZ800, Tokyo, Japan) with a fiber optic illuminator (NI‐150, Melville, NY, USA).

### 2.3 BP distribution analysis using stereomicroscopy

Tooth images were captured using NIS‐Elements software under the stereomicroscope with a fiber optic illuminator. Each image was deidentified and blind‐coded (VB) for the corresponding treatment information. The BP in root canals was analyzed based on two independent criteria of BP intensity and area. Lead author (VB) used ImageJ software (Schneider et al., 2012) to take measurements of blinded tooth images for BP area and intensity. The BP intensity was categorized into three levels, namely, no BP intensity (I0), low intensity (I1), and high intensity (I2). I0 was defined as no visible BP formation, I1 was defined as a light brown deposition formed due to a thin layer of BP and I2 was defined as a dark/intense brown deposition due to a thick layer of BP. The intensity grades were confirmed by co‐authors (NP and JWJ) who were also blinded to the group category of tooth images. The highest intensity grade in each tooth was selected for statistical analysis. The percent area of BP in the apical and middle one‐third of each tooth was calculated using the measure area tool in the ImageJ software (Schneider et al., 2012). Average % BP area was plotted for each treatment group. The coronal one‐third of the root was not analyzed due to an obscuring dentinal smear layer created by projected tooth debris off the diamond disc used during decoronation. It is likely that tooth positioning during decoronation limited the travel distance of projected smear layer debris to the coronal third only.

### 2.4 BP analysis using time‐of‐flight secondary ion mass spectrometry (ToF‐SIMS)

One tooth specimen per group was positioned with the cross‐sectioned root canal facing up. The sample holder attached to the transfer arm using a bayonet fitting was introduced in a chamber with a vacuum of 5.0 10‐6 mbar, ensuring an appropriate detection limit (few ppm). ToF‐SIMS analysis was performed using a ToF‐SIMS NCS instrument, which combines a ToF‐SIMS instrument (ION‐TOF GmbH, Münster, Germany) and an in‐situ VLS‐80 Scanning Probe Microscope (NanoScan, Switzerland). The probe was targeted on the shallow and flat area of the root canal wall at the middle one‐third of each root. High mass resolution spectra were collected. A Manta CCD camera (Allied Vision Technologies GmbH, Stadtroda, Germany) selected the most appropriate region in the root canal of each tooth specimen. For data analysis, each ion was normalized by total ion intensity to facilitate standardization and comparison between samples. The ion intensity from the regions of interest was calculated by extracting the area under the peak.

### 2.5 Statistical analysis of the BP intensity and area distribution

Teeth with BP intensity grades for each group were analyzed by Chi‐square test, generalized linear model (with binomial family) (*p* < .05) using R statistical Software (R Core Team, 2018). Percent BP area distribution for each group was analyzed using Tukey's multiple comparison test (*p* < .05). Group 1 and 2: *N* = 5; Group 3 and 4: *N* = 10; Group 5: *N* = 9.

## 3 RESULTS

### 3.1 Stereomicroscopy analysis shows a marked reduction in the BP with 5% STS

**Figure 1:**
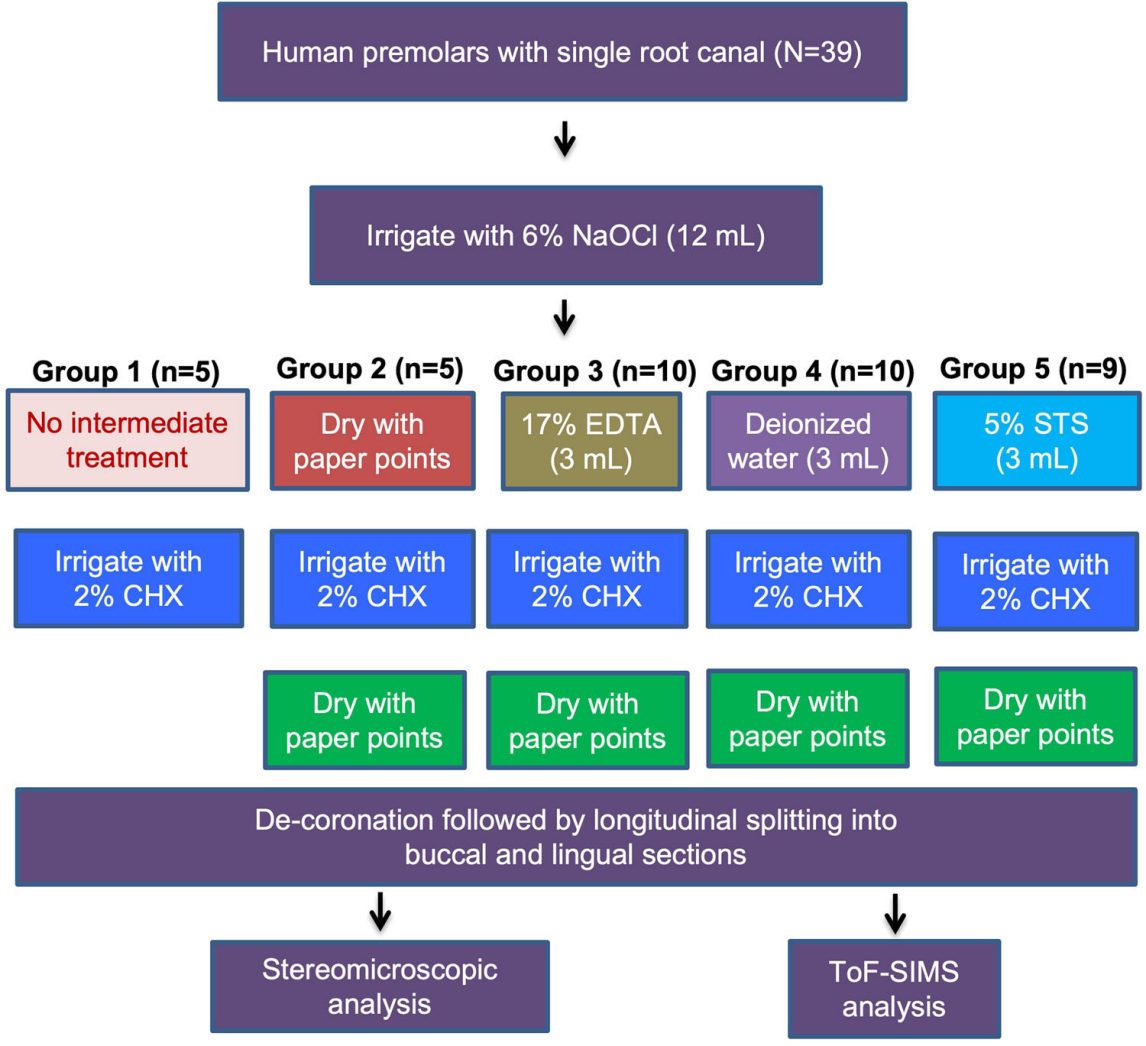
Schematic of different irrigation protocols tested in this study using extracted single‐rooted human premolars.

**Figure 2:**
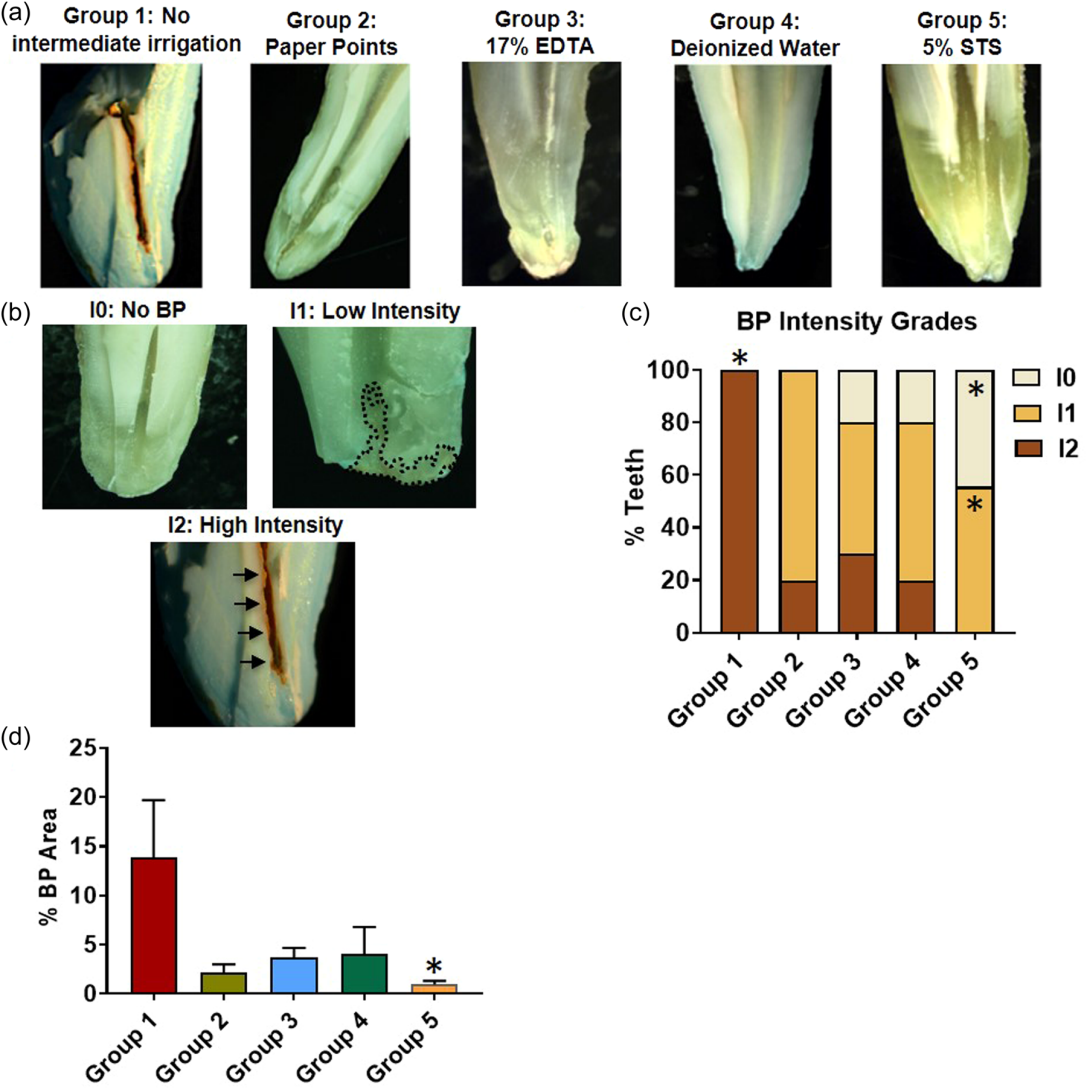
Stereomicroscopy‐based analysis shows a marked reduction in the BP with 5% STS. (a) Representative images for Groups 1–5 (b) Representative images for no (I0), low (I1, dotted area) or high (I2, arrows) BP intensity. (c) Percent teeth with BP intensity grades for Groups 1–5 (d) Percent BP area with SEM for Groups 1–5. *p < .05. BP, brown precipitate; EDTA, ethylenediaminetetraacetic acid; SEM, standard error of mean; STS, sodium thiosulfate

In a pilot in vitro assay, intermediate addition of 5% STS and 10% STS both prevented the BP formation as determined by a visually clear solution (data not shown). Due to equivalent efficacy, 5% STS was used in subsequent *ex vivo* tooth experiments. Extracted human premolars randomly assigned to Group 1, 2, 3, 4, or 5 underwent irrigation protocols as described in methods (Figure 1) followed by stereomicroscopy‐based analysis (Figure 2a). Representative images of BP distribution categorized for intensity levels are illustrated (Figure 2b). All teeth irrigated with 6% NaOCl and 2% CHX (Group 1, *N* = 5) showed high intensity (I2) BP (*p* < .001). In contrast, none of the teeth irrigated with 5% STS showed high intensity (I2) (*p* = .03) and significantly high percentage of teeth showed no BP (I0) (Group 5, *N* = 4/9, *p* = .04). Several teeth treated with either paper points (Group 2, *N* = 4/5), 17% EDTA (Group 3, *N* = 5/10) or deionized water (Group 4, *N* = 6/10) showed low‐intensity BP (I1) in root canals but did not reach statistical significance (Figure 2c, Group 2: *p* = .17, Group 3: *p* = .93, Group 4, *p* = .52). Compared with Group 1 with no intermediate treatment, teeth irrigated with 5% STS significantly reduced % BP area in root canals (Figure 2d, *p* = .01). Other treatment groups (Groups 2–4) showed reduced % BP area but not significantly different from Group 1. (Figure 2d, Groups 2 and 3: *p* = .06, Group 4: *p* = .07).

### 3.2 ToF‐SIMS analysis of teeth shows 5% STS as most efficacious in preventing the BP

**Figure 3:**
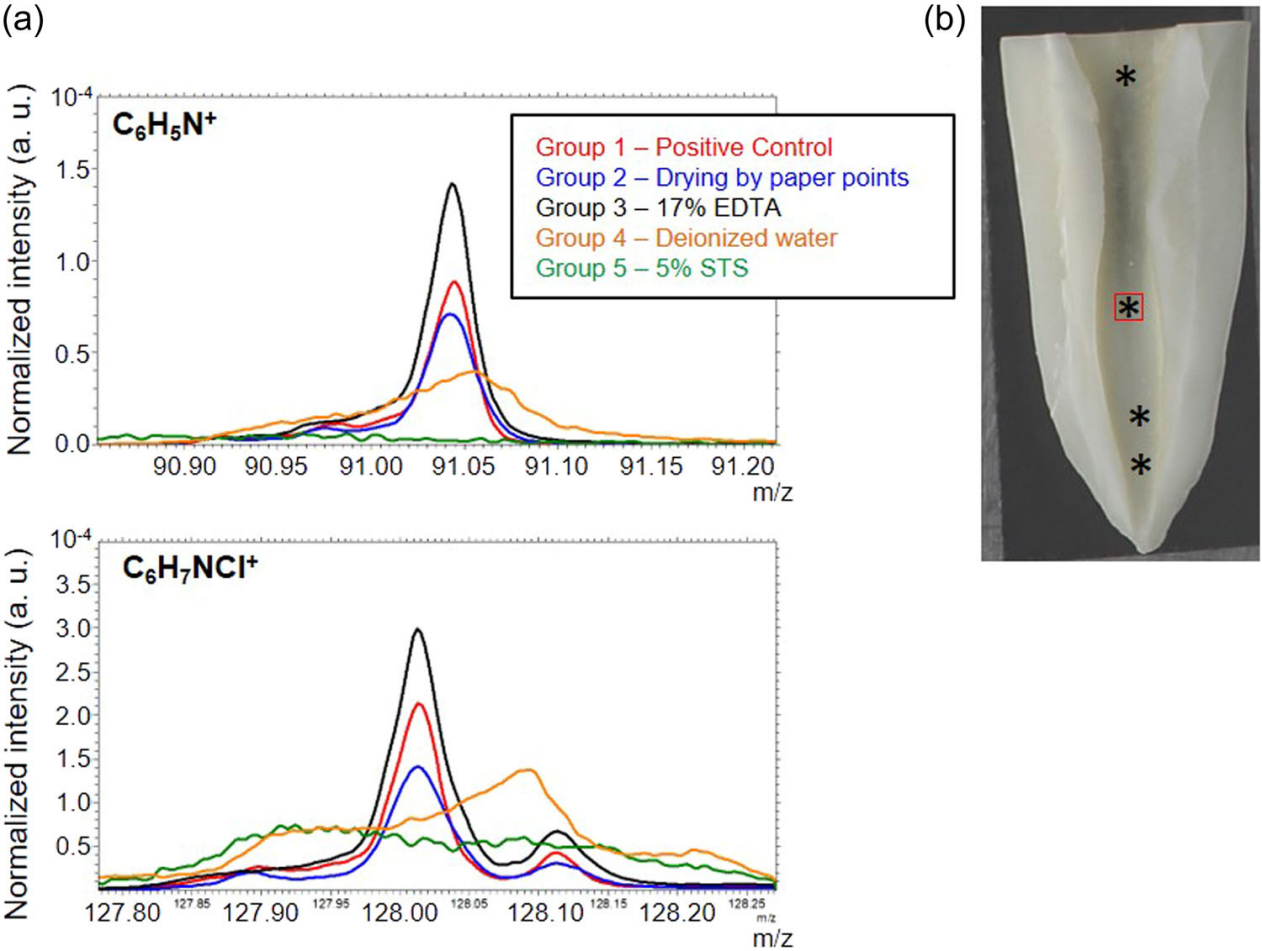
Intermediate irrigation with 5% STS reduces the detection of ions characteristic of CHX and para‐chloroaniline (a) Overlay of normalized positive ion ToF‐SIMS mass spectra of C_6_H_5_N^+^ and C_6_H_7_NCl^+^ in the root canal for Groups 1–5. (b) Representative image of a tooth positioned for ToF‐SIMS. Asterisks: data collection areas. Red square: Area of scan size around the wide and flat inner tooth surface selected for mass spectra analysis. ToF‐SIMS, time‐of‐flight secondary ion mass spectrometry; STS, sodium thiosulfate

ToF‐SIMS analysis of teeth treated consecutively with 6% NaOCl and 2% CHX (Group 1) detected C_6_H_5_N^+^ and C_6_H_7_NCl^+^ ions characteristic of para‐chloroaniline and CHX. Most reduction of the C_6_H_5_N^+^ and C_6_H_7_NCl^+^ was seen in teeth irrigated with 5% STS (Group 5) followed by deionized water (Group 4) and treatment with paper points (Group 2). 17% EDTA (Group 3) in contrast resulted in increased detection of the C_6_H_5_N^+^ and C_6_H_7_NCl^+^ (Table 1 and Figure 3a).

**Table 1:** ToF‐SIMS analysis of the extracted human premolars for ions representative of para‐chloroaniline and CHX

	Group 1	Group 2	Group 3	Group 4	Group 5
**C**_**6**_ **H**_**5**_ **N** ^**+**^	0.7790%	0.6580%	1.1700%	0.0180%	0.0095%
**C**_**6**_ **H**_**7**_ **NCl** ^**+**^	11.7000%	15.8000%	1.8000%	0.3730%	0.2600%

*Note*: Intensity of each positive ion was normalized to total positive ion intensity with area under the peak depicted for Groups 1–5.

Abbreviation: ToF‐SIMS, time‐of‐flight secondary ion mass spectrometry.

## 4 DISCUSSION

Because of the insoluble and toxic nature of BP (Bui et al., 2008; Cintra et al., 2014; Jeong et al., 2021; Nocca et al., 2017; Patil et al., 2016), it is important to investigate the appropriate prevention protocol in root canal treatments. Several intermediate irrigants have been suggested to prevent or reduce the formation of BP (Chhabra et al., 2018; Krishnamurthy & Sudhakaran, 2010; Mortenson et al., 2012). However, prior studies have relied on visual detection of BP. This is the first comparative study reporting the efficacy of 5% STS over other treatments to reduce BP using two independent approaches of stereomicroscopy and ToF‐SIMS. In this study, the stereomicroscopy analysis showed that intermediate irrigation with 5% STS eliminated high‐intensity BP (*p* < .05) whereas other intermediate treatments did not eliminate high‐intensity BP formation (Figure 2c). Intermediate irrigation with 5% STS also significantly reduced the % BP area (Figure 2d, *p* = .01). Results in this study agree with those of a recent study that reported prevention of BP following intermediate irrigation of bovine teeth with 5% STS using qualitative stereomicroscopic image analysis (Alberto et al., 2021). However, the previous study did not comparatively evaluate the efficacy of 5% STS with other intermediate treatments. In addition to quantitating BP intensity and area using stereomicroscopic image analysis, this study further examined the effect of intermediate treatments on BP formation using the ToF‐SIMS. Although this approach does not provide quantitative data, it facilitates comparative analysis of the relative distribution of ions within experimental groups. The C_6_H_5_N^+^ and C_6_H_7_NCl^+^ represent the components of para‐chloroaniline and CHX (Jeong et al., 2021). Like stereomicroscopy results, the ToF‐SIMS analysis showed that the intermediate irrigation with 5% STS most effectively reduced the detection of C_6_H_5_N^+^ and C_6_H_7_NCl^+^ (Figure 3a and Table 1). Interestingly, the ToF‐SIMS analysis of teeth treated with 17% EDTA as intermediate irrigant showed higher intensity of both C_6_H_5_N^+^ and C_6_H_7_NCl^+^ as compared with no intermediate treatment. This could either be due to a higher concentration of these ions formed with 17% EDTA or due to the matrix effect whereby the environment of the chemical compound promotes more ionization for the 17% EDTA treated samples as compared to other treatments. A limitation of the ToF‐SIMS results in this study was the decoronating procedure following irrigation, which spread tooth debris over the dentin lining the canal walls. This obstruction occurred due to the rotary diamond disc that, while cutting, produced a smear layer, which was projected into the canal and limited the number of suitable samples for ToF‐SIMS evaluation. Further limitations of this study were that the exact premolar root dimensions, that is, length, width, and thickness were not recorded before study. However, % BP area was recorded with respect to total tooth area, thus normalizing for variations in teeth size. Additionally, all groups were initially planned with equal number of human teeth, based on prior studies of comparable experimental design, and following consultation with a statistician (JNH). However, some teeth were discarded due to damage that occurred at different phases of the experiment This was likely because of the extraoral dry time of the human teeth ex‐vivo, and some teeth were notably more brittle than others. Based on clear preliminary results for Groups 1 and 2, and the limited availability of human teeth, the sample size in Groups 1 and 2 were limited compared to other experimental groups as described in methods. Lastly, it was not possible for us to follow and record if the BP formed following a specific irrigation protocol was in part dislodged from the canal walls during longitudinal sectioning. However, BP was shown to adhere to dentin and significantly occlude dentinal tubules (Bui et al., 2008). Therefore, it is unlikely that BP was dislodged during the longitudinal sectioning of samples.

## 5 CONCLUSIONS

This study shows that in comparison to deionized water, 17% EDTA or drying with paper points, 5% STS was more efficacious in eliminating BP and ions associated with CHX and para‐chloroaniline. These results confirm our hypothesis, that 5% STS is a more effective intermediate irrigation solution, as compared to 17% EDTA, deionized water, or drying with paper points in preventing the formation of BP.

## AUTHOR CONTRIBUTIONS

**Vashti Bueso**: Conceptualization; methodology; data collection and analysis; writing—original draft, review and editing; project administration. **Neha Parikh**: Conceptualization; data analysis; writing—original draft, review and editing; supervision. **Tanguy Terlier**: methodology; data collection and analysis, writing—original draft. **Julian N Holland**: Statistical analysis. **Nima Sarmast**: Conceptualization; writing—review and editing. **Ji Wook Jeong**: Conceptualization; methodology; data analysis; writing—original draft; review and editing; supervision

## CONFLICTS OF INTEREST

The authors declare no conflicts of interest.

## AUTHORSHIP DECLARATION

All authors have contributed significantly, and all authors are in agreement with the manuscript.

## Data Availability

The data that support the findings of this study are available from the corresponding author upon reasonable request.

## References

[cre2654-bib-0001] Alberto, A. P. L. , Oliveira, D. D. S. , Oliveira, H. E. , Maciel, A. C. C. , Belladonna, F. G. , & Silva, E. (2021). Does sodium thiosulphate avoid the formation of the brown‐coloured precipitate as an intermediate irrigant between NaOCl and chlorhexidine? Australian Endodontic Journal, 48, 72–76. 34494676 10.1111/aej.12562

[cre2654-bib-0002] Basrani, B. R. , Manek, S. , Mathers, D. , Fillery, E. , & Sodhi, R. N. (2010). Determination of 4‐chloroaniline and its derivatives formed in the interaction of sodium hypochlorite and chlorhexidine by using gas chromatography. Journal of Endodontics, 36, 312–314. 20113798 10.1016/j.joen.2009.10.031

[cre2654-bib-0003] Basrani, B. R. , Manek, S. , Sodhi, R. N. , Fillery, E. , & Manzur, A. (2007). Interaction between sodium hypochlorite and chlorhexidine gluconate. Journal of Endodontics, 33, 966–969. 17878084 10.1016/j.joen.2007.04.001

[cre2654-bib-0004] Bui, T. B. , Baumgartner, J. C. , & Mitchell, J. C. (2008). Evaluation of the interaction between sodium hypochlorite and chlorhexidine gluconate and its effect on root dentin. Journal of Endodontics, 34, 181–185. 18215677 10.1016/j.joen.2007.11.006

[cre2654-bib-0005] Chhabra, N. , Gangaramani, S. , Singbal, K. P. , Desai, K. , & Gupta, K. (2018). Efficacy of various solutions in preventing orange‐brown precipitate formed during alternate use of sodium hypochlorite and chlorhexidine: An in vitro study. Journal of Conservative Dentistry: JCD, 21, 428–432. 30122826 10.4103/JCD.JCD_72_18 PMC6080170

[cre2654-bib-0006] Chhabra, R. S. , Huff, J. E. , Haseman, J. K. , Elwell, M. R. , & Peters, A. C. (1991). Carcinogenicity of p‐chloroaniline in rats and mice. Food and Chemical Toxicology, 29, 119–124. 2010141 10.1016/0278-6915(91)90166-5

[cre2654-bib-0007] Cintra, L. T. , Watanabe, S. , Samuel, R. O. , da Silva Facundo, A. C. , de Azevedo Queiroz, I. O. , Dezan‐Júnior, E. , & Gomes‐Filho, J. E. (2014). The use of NaOCl in combination with CHX produces cytotoxic product. Clinical Oral Investigations, 18, 935–940. 23892500 10.1007/s00784-013-1049-5

[cre2654-bib-0008] Grawehr, M. , Sener, B. , Waltimo, T. , & Zehnder, M. (2003). Interactions of ethylenediamine tetraacetic acid with sodium hypochlorite in aqueous solutions. International Endodontic Journal, 36, 411–417. 12801288 10.1046/j.1365-2591.2003.00670.x

[cre2654-bib-0009] Hegde, J. , Bashetti, K. , Krishnakumar, K. , & Gulati, U. (2012). Quantity of sodium thiosulfate required to neutralize various concentrations of sodium hypochlorite. Asian Journal of Pharmaceutical Sciences, 2, 390–393.

[cre2654-bib-0010] Jeong, J. W. , Sarmast, N. D. , Terlier, T. , van der Hoeven, R. , Holland, J. N. , & Parikh, N. (2021). Assessment of the cytotoxic effects and chemical composition of the insoluble precipitate formed from sodium hypochlorite and chlorhexidine gluconate. International Endodontic Journal, 54, 1892–1901. 34081782 10.1111/iej.13583

[cre2654-bib-0011] Krishnamurthy, S. , & Sudhakaran, S. (2010). Evaluation and prevention of the precipitate formed on interaction between sodium hypochlorite and chlorhexidine. Journal of Endodontics, 36, 1154–1157. 20630289 10.1016/j.joen.2010.01.012

[cre2654-bib-0012] Möller, A. J. (1966). Microbiological examination of root canals and periapical tissues of human teeth. Methodological studies. Odontologisk Tidskrift, 74(Suppl), 1–380. 5335186

[cre2654-bib-0013] Mortenson, D. , Sadilek, M. , Flake, N. M. , Paranjpe, A. , Heling, I. , Johnson, J. D. , & Cohenca, N. (2012). The effect of using an alternative irrigant between sodium hypochlorite and chlorhexidine to prevent the formation of para‐chloroaniline within the root canal system. International Endodontic Journal, 45, 878–882. 22486894 10.1111/j.1365-2591.2012.02048.x

[cre2654-bib-0014] Naenni, N. , Thoma, K. , & Zehnder, M. (2004). Soft tissue dissolution capacity of currently used and potential endodontic irrigants. Journal of Endodontics, 30, 785–787. 15505511 10.1097/00004770-200411000-00009

[cre2654-bib-0015] Nocca, G. , Ahmed, H. M. A. , Martorana, G. E. , Callà, C. , Gambarini, G. , Rengo, S. , & Spagnuolo, G. (2017). Chromographic analysis and cytotoxic effects of chlorhexidine and sodium hypochlorite reaction mixtures. Journal of Endodontics, 43, 1545–1552. 28734651 10.1016/j.joen.2017.04.025

[cre2654-bib-0016] Patil, P. , Aminoshariae, A. , Harding, J. , Montagnese, T. A. , & Mickel, A. (2016). Determination of mutagenicity of the precipitate formed by sodium hypochlorite and chlorhexidine using the Ames test. Australian Endodontic Journal, 42, 16–21. 25612244 10.1111/aej.12100

[cre2654-bib-0017] Pimentel Corrêa, A. C. , Cecchin, D. , de Almeida, J. F. , Gomes, B. P. , Zaia, A. A. , & Ferraz, C. C. (2016). Sodium thiosulfate for recovery of bond strength to dentin treated with sodium hypochlorite. Journal of Endodontics, 42, 284–288. 26723482 10.1016/j.joen.2015.11.010

[cre2654-bib-0018] R Core Team (2018). R: A language and environment for statistical computing. R Foundation for Statistical Computing V, Austria. https://www.R-project.org/

[cre2654-bib-0019] Sariyilmaz, E. , Sivas Yilmaz, Ö. , Keskin, C. , & Keleş, A. (2019). Effect of sodium hypochlorite and chlorhexidine irrigating solutions and their inactivating agents on the push‐out bond strength of mineral trioxide aggregate. Biomedical Materials and Engineering, 30, 279–285. 30988236 10.3233/BME-191051

[cre2654-bib-0020] Schneider, C. A. , Rasband, W. S. , & Eliceiri, K. W. (2012). NIH Image to ImageJ: 25 years of image analysis. Nature Methods, 9, 671–675. 22930834 10.1038/nmeth.2089 PMC5554542

[cre2654-bib-0021] Yesilsoy, C. , Whitaker, E. , Cleveland, D. , Phillips, E. , & Trope, M. (1995). Antimicrobial and toxic effects of established and potential root canal irrigants. Journal of Endodontics, 21, 513–515. 8596073 10.1016/s0099-2399(06)80524-8

[cre2654-bib-0022] Zehnder, M. (2006). Root canal irrigants. Journal of Endodontics, 32, 389–398. 16631834 10.1016/j.joen.2005.09.014

